# Spirulina Promotes Stem Cell Genesis and Protects against LPS Induced Declines in Neural Stem Cell Proliferation

**DOI:** 10.1371/journal.pone.0010496

**Published:** 2010-05-05

**Authors:** Adam D. Bachstetter, Jennifer Jernberg, Andrea Schlunk, Jennifer L. Vila, Charles Hudson, Michael J. Cole, R. Douglas Shytle, Jun Tan, Paul R. Sanberg, Cyndy D. Sanberg, Cesario Borlongan, Yuji Kaneko, Naoki Tajiri, Carmelina Gemma, Paula C. Bickford

**Affiliations:** 1 Department of Molecular Pharmacology and Physiology, College of Medicine, University of South Florida, Tampa, Florida, United States of America; 2 Department of Neurosurgery and Brain Repair, Center of Excellence for Aging and Brain Repair, College of Medicine, University of South Florida, Tampa, Florida, United States of America; 3 James A. Haley Veterans Administration Hospital, Research Service, Tampa, Florida, United States of America; 4 Department of Psychiatry, College of Medicine, University of South Florida, Tampa, Florida, United States of America; 5 NaturaTherapeutics, Incorporated, Tampa, Florida, United States of America; University of Nebraska Medical Center, United States of America

## Abstract

Adult stem cells are present in many tissues including, skin, muscle, adipose, bone marrow, and in the brain. Neuroinflammation has been shown to be a potent negative regulator of stem cell and progenitor cell proliferation in the neurogenic regions of the brain. Recently we demonstrated that decreasing a key neuroinflammatory cytokine IL-1β in the hippocampus of aged rats reversed the age-related cognitive decline and increased neurogenesis in the age rats. We also have found that nutraceuticals have the potential to reduce neuroinflammation, and decrease oxidative stress. The objectives of this study were to determine if spirulina could protect the proliferative potential of hippocampal neural progenitor cells from an acute systemic inflammatory insult of lipopolysaccharide (LPS). To this end, young rats were fed for 30 days a control diet or a diet supplemented with 0.1% spirulina. On day 28 the rats were given a single i.p. injection of LPS (1 mg/kg). The following day the rats were injected with BrdU (50 mg/kg b.i.d. i.p.) and were sacrificed 24 hours after the first injection of BrdU. Quantification of the BrdU positive cells in the subgranular zone of the dentate gyrus demonstrated a decrease in proliferation of the stem/progenitor cells in the hippocampus as a result of the LPS insult. Furthermore, the diet supplemented with spirulina was able to negate the LPS induced decrease in stem/progenitor cell proliferation. In a second set of studies we examined the effects of spirulina either alone or in combination with a proprietary formulation (NT-020) of blueberry, green tea, vitamin D3 and carnosine on the function of bone marrow and CD34^+^ cells *in vitro*. Spirulina had small effects on its own and more than additive effects in combination with NT-020 to promote mitochondrial respiration and/or proliferation of these cells in culture. When examined on neural stem cells in culture spirulina increased proliferation at baseline and protected against the negative influence of TNFα to reduce neural stem cell proliferation. These results support the hypothesis that a diet enriched with spirulina and other nutraceuticals may help protect the stem/progenitor cells from insults.

## Introduction

Neurogenesis is a life-long occurrence that is limited to specific sites within the brain; namely, the subventricular zone (SVZ), and the subgranular zone (SGZ) of the hippocampus. The *de novo* production of new neurons into the hippocampus, has been shown to be important for some forms of learning [1]. While numerous studies have shown that neurogenesis is physiologically relevant for cognitive function, the relationship is complex (For review see: [2], [3]). Nonetheless, neurogenesis is clearly linked to plasticity and repair mechanisms [4] and alterations in neurogenesis have been also been attributed to some affective disorders [5].

Two seminal studies, published simultaneously, a number of years ago showed that inflammation tightly regulates neurogenesis in the SGZ [6], [7]. Ekdahl et al. (03) used LPS that they delivered into the cortex continuously by an osmotic mini pump. In the young adult rat, LPS-induced inflammation resulted in an 85% reduction in the number of new neurons born during the inflammatory insult [6]. Monje et al. (03) also found that LPS given systemically caused an increase in microglia activation and a decrease in neurogenesis, which could be prevented by the nonsteroidal anti-inflammatory drug (NSAID) indomethacin [7].

Cytokines do appear capable of regulating several phases of the neurogenesis process. At low concentrations the proinflammatory cytokine TNF-α induces proliferation of neural progenitor cells (NPC), but at higher concentrations TNF-α results in program cell death [8]. TNF-α induced program cell death in the NPC is dependent on TNF receptor 1 (TNF-RI) [9] which is constitutively expressed by NPC in culture [10]. IL-1β can also directly suppress neurogenesis by blocking the production of cyclic dependent kinesis [10], [11]. Inflammation also alters the way the new neurons integrate into the existing neuronal circuit [12].

We have previously demonstrated that spirulina can act as an anti-inflammatory, and reverse the age-related elevation of TNF-α [13]. Activation of TNF-RI by TNF-α negatively regulates the proliferation of NPC [10]. Therefore we hypothesized that spirulina could protect against an acute systemic inflammatory insult induced by LPS the effects would occur during the proliferative phase of neurogenesis. To test this hypothesis, young adult Fisher 344 rats were fed a control diet, or a diet supplemented with 0.1% spirulina for 28 days before administration of LPS. As predicted, LPS decreased proliferation of the hippocampal NPCs. Spirulina was able to prevent the LPS induced decrease in NPC proliferation. The LPS insult did not appear to have pronounced affects on microglial activation but did produce a measurable increase in astrogliosis. Pretreatment with spirulina also blocked the LPS induced astrogliosis. The *in vivo* results suggest that spirulina protects by a non-stem cell autonomous mechanisms.

Previous studies have shown that nutraceuticals can have effects on adult stem cells. A nutraceutical combination of blueberry extract, green tea extract, carnosine, and vitamin D3 (a proprietary formulation known as NT-020) has been shown to promote migration of brain stem cells from the stem cell niche to the site of injury in an animal model of stroke [4]. NT-020 was shown to stimulate the proliferation of human stem cells derived from bone marrow; bone marrow derived CD34^+^ and progenitor cells from peripheral blood (CD133^+^) *in vitro* [14]. Cultured bone marrow cells removed from mice given NT-020 orally for 2 weeks exhibited a dose-related reduction of oxidative stress-induced cell death. This demonstrates that the action of this nutraceutical on stem cells is not dependent on the presence of the formulation as the effect was observed when the cells were cultured in the absence of NT-020 for 3 days.

To investigate if spirulina could promote proliferation of cells other than neural stem cells investigated the effects of spirulina both alone and in combination with NT-020 to promote the proliferation of bone marrow and CD34^+^ stem cells in culture as measured with MTT. We found that spirulina increased proliferation/mitochondrial respiration of CD34^+^ stem cells in culture. The results of this study demonstrate that spirulina can negate the negative effects of inflammation on neurogenesis, indirectly by decreasing astrogliosis and by potentially acting directly on the stem cells and promote proliferation of bone marrow and CD34+ human stem cells in culture.

## Results

### Spirulina protects against LPS induced decrease in hippocampal progenitor cell proliferation

**Figure 1:**
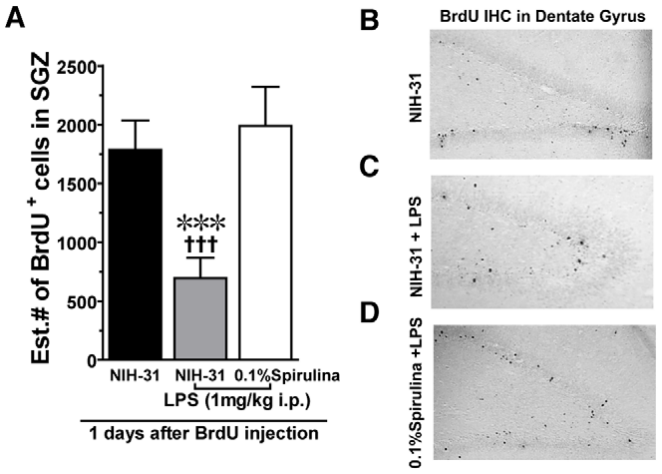
Spirulina protects against LPS induced decrease in cell proliferation. (**A**) Results of the Stereological counts of the number of BrdU^+^ cells demonstrated that spirulina was able completely block the LPS induced decline in inflammation. *** p = 0.0049 (NIH-31 vs. NIH-31+LPS). ††† P = 0.0090 (NIH-31+LPS vs. spirulina+LPS). (**B**) Shows the representative BrdU staining in the rats without LPS. (**C**) After LPS in the rats fed the control diet there are fewer BrdU^+^ cells. (**D**) In the spirulina fed rat LPS did not cause a decrease in proliferation. (All photomicrographs taken at 10× magnification).

Before induction of the LPS inflammatory insult, young adult male Fisher 344 rats were fed either a standard NIH-31 diet or a spirulina-enriched diet (NIH-31 +0.1% spirulina). After 28 days on the diets the rats were injected with LPS (1 mg/kg) into the peritoneal cavity to induce an acute inflammatory insult. The following day, day 29, the rats were injected twice (8 hours interval) with BrdU (50 mg/kg) to label those cells that were proliferating while BrdU was available. The rats were then euthanized on the following day to determine if spirulina could protect against the anti-neurogenic effects of LPS (see Fig. 1A for timeline). Quantification of the BrdU labeled cells, in the SGZ of the hippocampus, was performed using the optical fractionator method of design based stereology [15]. A significant effect was found (F_(2,19)_ = 5.913, p<0.05) in the number of BrdU^+^ cells in the SGZ in the rats euthanized two days after the inflammatory insult of LPS (Fig. 1A). As we predicted, LPS did significantly decrease the number of BrdU^+^ cells in the rats fed the control diet (t_(10)_ = 3.589; p = 0.0049). The cytokine storm induced by LPS peaks 2 hours after intraperitoneal injection of LPS [16], we found that even one full day after the peak of the cytokine storm, proliferation of the NPC was reduced by nearly 40% in rats fed the control diet. A diet enriched with spirulina was able to completely prevent the LPS induced decrease in NPC proliferation. In rats fed a spirulina enriched diet prior to being injected with LPS, proliferation of NPC was not significantly different then the control fed rats that were not injected with LPS. Moreover, in comparison to the control fed rats that were injected with LPS, the spirulina fed rats that were also injected with LPS had significantly more BrdU labeled cells in the SGZ (t_(12)_ = 3.113; p = 0.0090).

**Figure 2:**
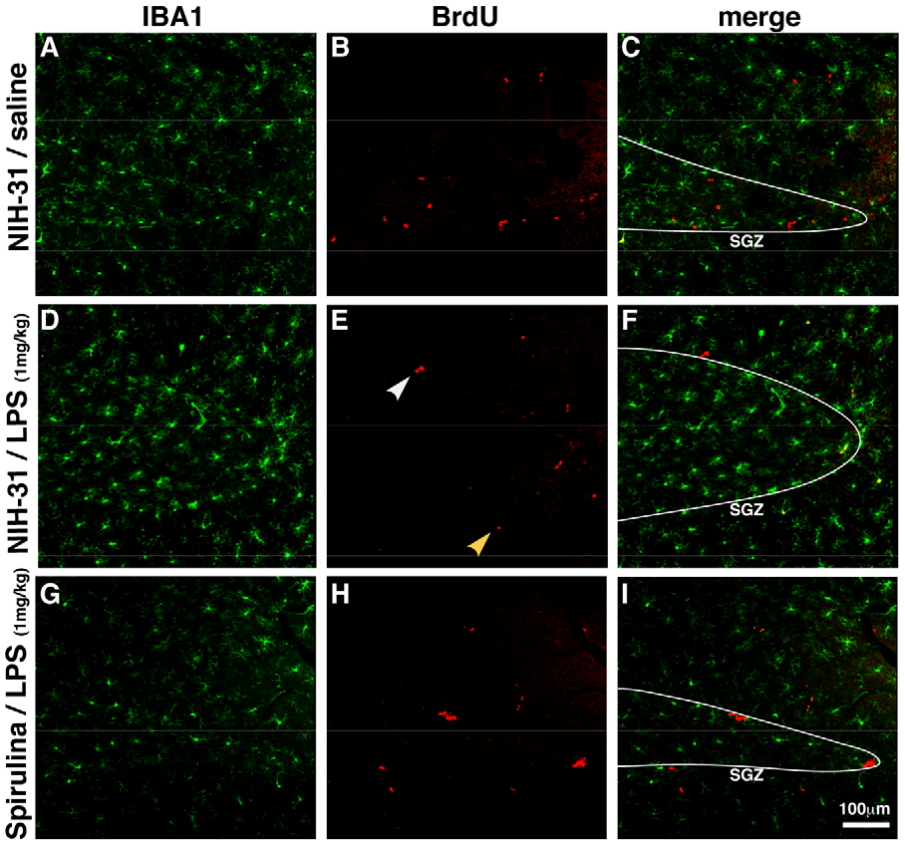
BrdU found in microglia only in the control fed mice following LPS. Low magnification confocal photomicrographs were used to determine if the BrdU was labeling non-neural cells. (**A–C**) In the control animals without LPS the majority of the BrdU cells were not found to be microglia. (**D–E**) In the control diet fed rats treated with LPS note that fewer BrdU cells were found and that many of the BrdU labeled cells were microglia. White arrow shown in high magnification in Fig. 3D–F Yellow arrow shown in high magnification in Fig. 3G–I. (**G–I**) In the spirulina fed rats the BrdU cells were not found to be microglia.

**Figure 3:**
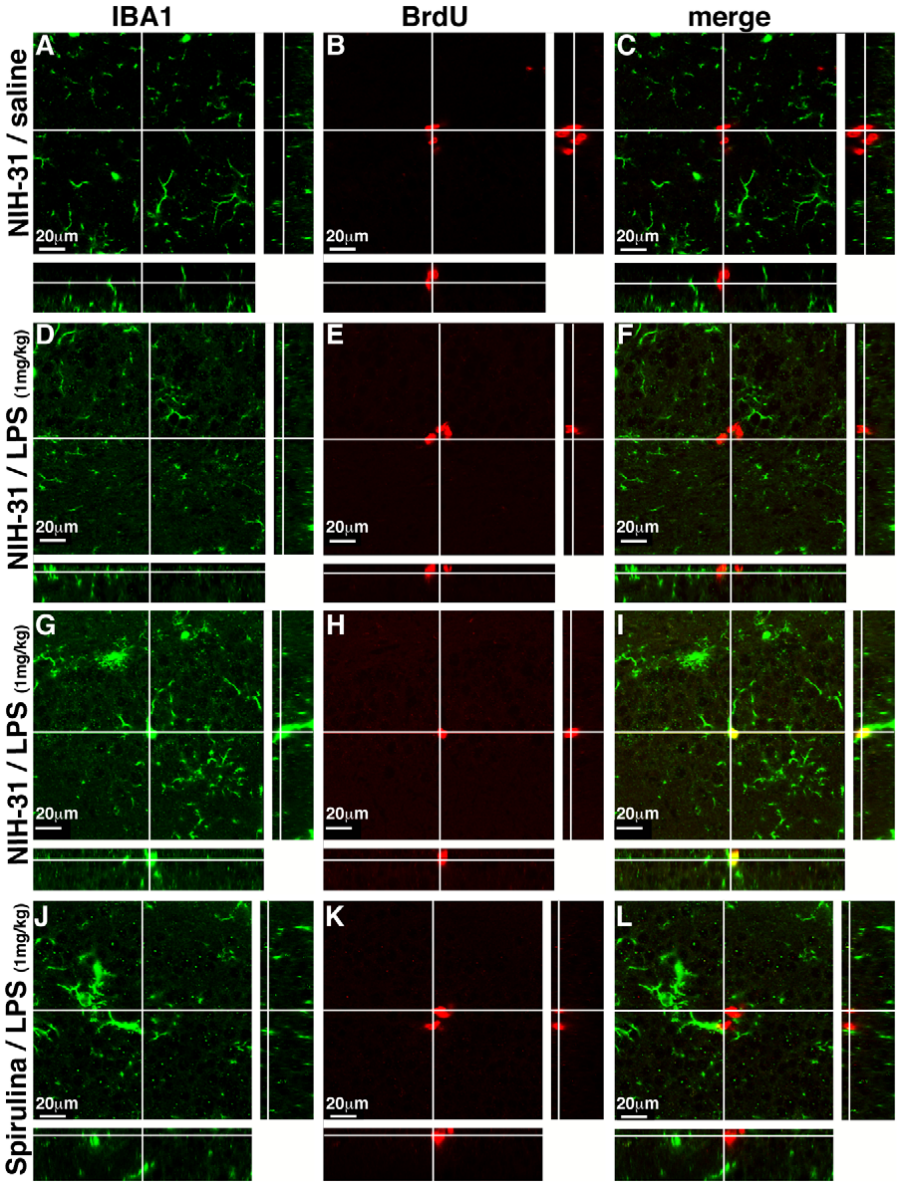
Orthogonal projections of BrdU IBA1 co-staining. (**A–C**) shows a representative cluster of BrdU^+^ cells in the SGZ confirming the cells are not microglia by the lack of colocalization with IBA-1. (**D–E**) shows a cluster of BrdU^+^ cells in the LPS treated rats that are not microglia. (**G–I**) shows a BrdU^+^ that does double label with the microglia marker IBA-1 as noted by the yellow color in the merged panel. (**J–K**) shows BrdU and IBA-1 staining in the Spirulina fed rats. Note, in this and all confocal images the side panels for each box are 90° rotations of the Z-stacks for confirmation of double labeling.

The incorporation of BrdU in the DNA can occur in any cell that is actively synthesizing DNA. While incorporation of BrdU can occur in cells that are repairing DNA, it has been demonstrated that the amount of BrdU added to DNA during the repair process is typically below the level necessary to be detected by standard immunohistochemistry procedures [17]. BrdU does not discriminate proliferating neurons or glia. As LPS could increase the proliferation of glia, particularly microglia, confocal microscopy was used to determine if the BrdU was labeling glia. Figure 2 and 3, show the photomicrographs of BrdU (red) and Iba-1 (green). Iba-1 is a calcium-binding protein that is restrictedly expressed in macrophages/microglia [18]. In the neurogenic region of the hippocampus, BrdU+ cells were not found to be double labeled with Iba-1 to any measurable extent in control fed rats without LPS or in spirulina fed rats with LPS. However, in the control fed rats that were injected with LPS some BrdU^+^ cells were found in the SGZ that were double-labeled with Iba-1 (Fig. 2D–E, Fig. 3G–I).

**Figure 4:**
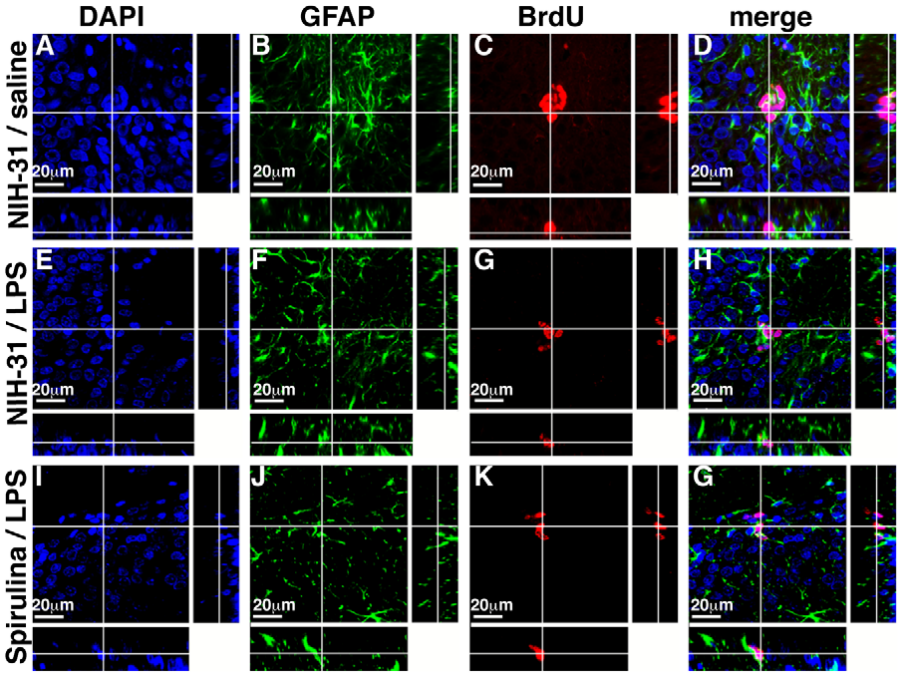
Orthogonal projections of BrdU GFAP co-staining. (**A–D**) Confocal images with orthogonal views demonstrate the absence of co-labeling of BrdU and GFAP in the 4 groups of animals examined. In the merged column you can observe co-localization of BrdU with DAPI as indicated by the purple. In constrast there is no colocalization of BrdU with GFP although the GFP labeled cells are closely associated with the BrdU positive cells. This supports our finding of no increased absolute numbers of GFAP labeled cells between any of the groups examined.

The stem cell/progenitor cell in the brain is proposed to be of an astrocyte lineage. However, the GFAP^+^ stem cell is less proliferative then the transient amplifying cell, and normally accounts for a small percentage of the proliferating cells. Figure 4 shows photomicrographs of BrdU (red) and GFAP (green) immunofluorescence staining. In the all three groups very few of the BrdU+ cells were found to be double labeled with GFAP. There did not appear to be any differences between groups in the number of BrdU^+^/GFAP^+^ cells; however, the relative scarcity of double labeled cells made it impossible to make reliable statistical comparisons between groups.

### Spirulina does not alter microglia response to LPS

The expression of MHC Class II on microglia is not constitutive but is inducible upon microglia activation. The number of cells expressing MHC Class II was quantified using the marker OX-6. Initial analysis of OX-6 expression demonstrated relatively few OX-6^+^ cells in any of the treatment groups. To limit errors due to under-sampling the entire hippocampus was used as the area sampling fraction. In rats that did not receive LPS there was no detectable OX-6^+^ cells in the hippocampal parenchyma. While still rare, OX-6^+^ cells were seen in association with blood vessels, indicating that the lack of OX-6^+^ cells was not due to poor staining. In the LPS treat rats, OX-6+ cells were found in the hippocampal parenchyma; however, there was still very few OX-6^+^ cell, resulting in large amount of intra group variability. No statistical difference was found in the rats that received LPS and fed either a diet enriched with spirulina or a control diet (data not shown).

### Spirulina protects from LPS induced astrogliosis

**Figure 5:**
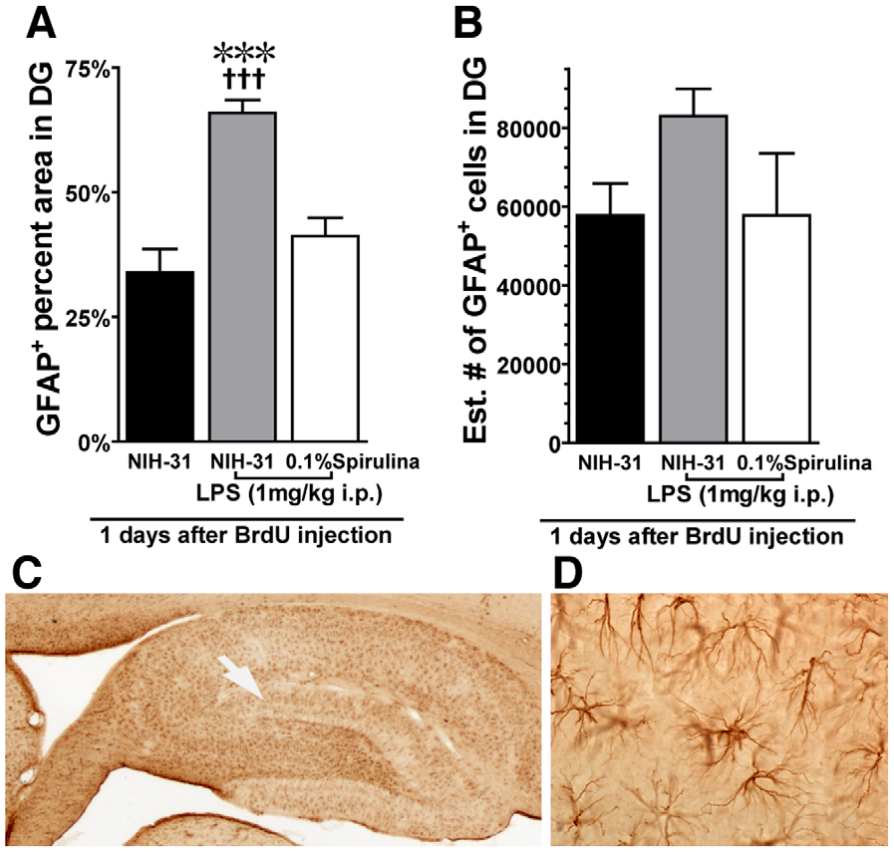
Spirulina rats have a decreased astrocyte response to LPS. (**A**) LPS caused a significant increase in the percentage of the dentate gyrus which was GFAP positive. The spirulina fed rats had a reduced GFAP response LPS compare to the control diet fed rats with LPS. *** p = 0.0002 (NIH-31 vs. NIH-31+LPS). ††† P = 0.0003 (NIH-31+LPS vs. spirulina+LPS). (**B**) There was no significant difference in the total number of GFAP^+^ cells. (**C**) Representative photomicrograph of the GFAP immunohistochemical staining in the hippocampus. The white arrow points to the area shown in (**D**) at 20× magnification.

Astrocyte activation has been demonstrated to accompany a neuronal insult [19]. Elevation in the expression of glial fibrillary acidic protein (GFAP) has been used to demonstrate an increase in activated astrocytes. Increased GFAP immunoreactivity is believed to occur due to increased expression on existing cells and not due to an increase in the number of astrocytes. To quantify the expression of GFAP in the dentate gyrus we used the design-based stereological probe, area fraction fractionator. Quantification of the percentage of the dentate gyrus that was immunoreactive for GFAP revealed a significant effect of treatment (F_(2, 14)_ = 24.04, p<0.0001; Figure 5.A). LPS induced a significant increase in percentage of the dentate gyrus that was occupied by GFAP immunoreactivity in control diet fed rats (t_(8)_ = 6.496; p = 0.0002). Spirulina was able to prevent the LPS induced increase GFAP immunoreactivity. There was no significant difference between the rats that were control rats that did not receive LPS and the spirulina fed rats that did receive LPS. Moreover, the spirulina fed rats that received the LPS insult had significant less astrogliosis then the matched control rats that received LPS (t_(9)_ = 5.683; p = 0.0003). Using the optical fractionator method of design based stereology we also quantified the total number of GFAP^+^ cells in the dentate gyrus. While there was a significant increase in the area of the dentate gyrus that was GFAP positive there was not a significant effect in the number of GFAP^+^ astrocytes following the LPS induced inflammatory insult (Figure 5.B). Therefore after the LPS insult the increase in astrogliosis was due to an increase activation of GFAP in existing cells. In support that there was not an increase in the number of astrocytes we did not find an increase in GFAP^+^/BRDU^+^ cells in the dentate gyrus in any of the groups (Fig. 4). The non-significant increase in the number GFAP^+^ cells in the dentate gyrus of the LPS treated control fed rats is most likely a result of an increase in GFAP expression in cells that would have otherwise been undetectable.

### Spirulina increases proliferation/mitochondrial function of human stem cells in culture

**Figure 6:**
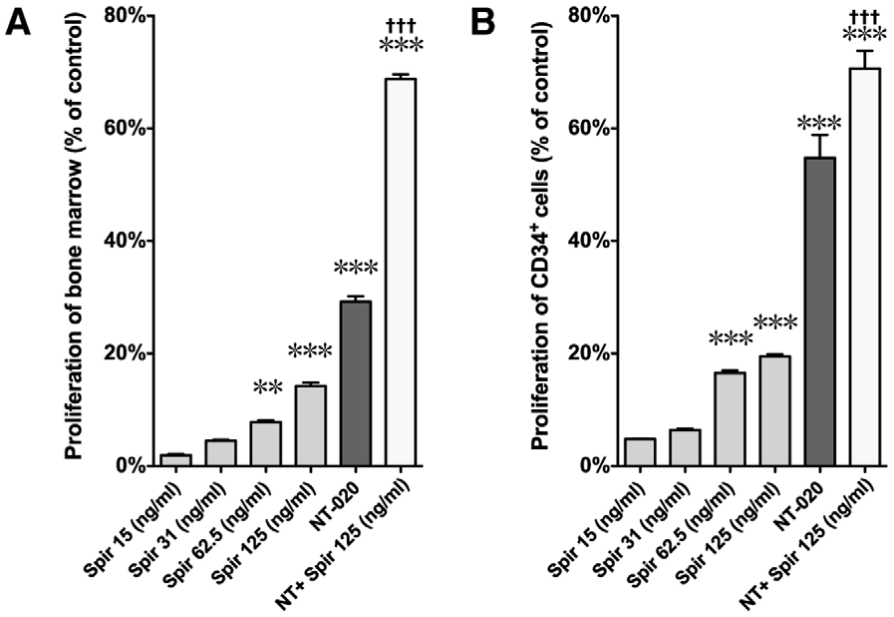
Spirulina increases proliferation of human hematopoetic stem cells *in vitro*. (**A**) The effects of spirulina and NT-020 on the proliferation of human bone marrow cells was examined. Cells were plated in 96 well plates and spirulina at varying concentrations was added to the culture media. In a separate study shown in the same graph the highest dose of spirulina tested alone (125 ng/ml) was added to NT-020. After 72 hours in culture viability was tested using the MTT assay. Data is expressed at % over control, which are cells grown in media only. When spirulina is combined with NT-020 the effect is significantly higher than NT-020 alone was more than additive. (**B**) The effects of spirulina and NT-020 on the proliferation of human CD34^+^ cells was examined. Cells were plated in 96 well plates and spirulina at varying doses was added to the culture media, in a separate study shown in the same graph the highest dose of spirulina tested alone (125 ng/ml) was added to NT-020. After 72 hours in culture viability was tested using the MTT assay. Data is expressed at % over control, which is cells grown in media only. When spirulina is combined with NT-020 the effect is significantly higher than NT-020 alone (p<0.05 students 2-tailed t-test) and the effect appears to be additive. ** p<0.001 ***p<0.0001 (compare to control). P<0.0001 (NT-020 vs. spirulina+NT-020).

We analyzed the effects of spirulina on human neural progenitor cells and human bone marrow and CD34^+^ cells in culture to determine if spirulina could have a potentially direct effect on the proliferation of stem cells. Using this assay we previously found that NT-020 could promote proliferation of bone marrow and bone marrow derived CD34^+^ progenitors and progenitor cells from peripheral blood (CD133^+^) *in vitro* [14]. Using this previously validated assay, human bone marrow cells were grown in culture with either spirulina alone, NT-020 alone, or spirulina in combination with NT-020 added to the culture media for 72 hours. A significant effect was found in the bone marrow cells proliferation assay (F_(5,35)_ = 166.8, p<0.0001; Fig 6.A). Replicating previously published results, NT-020 significantly increased proliferation of bone marrow cells (p<0.001; Fig 6A) [14]. Spirulina alone at concentrations of 62 ng/ml (p<0.001) and 125 ng/ml (p<0.001) significantly increased proliferation of bone marrow cells in culture when compared with control conditions without spirulina. When spirulina (125 ng/ml) was combined with NT-020 the combined group was significantly different from NT-020 alone (p<0.001). Moreover the effect on cell proliferation was 50% greater then what would be expected if the increase in proliferation was an additive effect. One caveat to take into consideration when considering these results is that the MTT assay measures mitochondrial function and thus is an indirect measure of proliferation, thus if a primary effect of these treatments is an increase in mitochondrial respiration, then this may exaggerate the conclusion of proliferative effects of this treatment.

Bone marrow cells are a mixed population of stem cells, immature and mature hematopoietic cells, thus we also examined the effect of spirulina on isolated human peripheral blood CD34^+^ stem cells (Fig. 6.B). Replicating the effects of the bone marrow cells, a significant effect was found in the CD34^+^ cells in the proliferation assay (F_(5,27)_ = 166.8, p<0.0001; Fig 6.B). At concentrations of spirulina 62 ng/ml or higher significant increase in proliferation of CD34^+^ stem cells in culture was found (p<0.001). When examined in combination with NT-020 there was also an additive effect of spirulina (125 ng/ml) on NT-020 in the CD34^+^ stem cells p<0.001).

**Figure 7:**
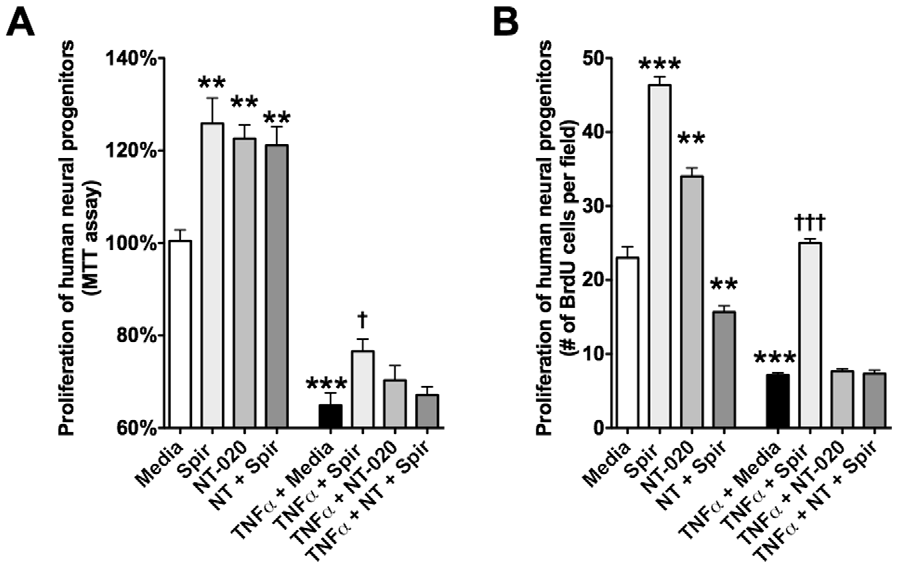
Spirulina increases proliferation of human neural stem cells *in vitro* and protects against a TNFα insult. Human neural progenitors grown under proliferation conditions were assessed by MTT assay (**A**) or BrdU (**B**) for the effects of spirulina (125 ng/ml) or NT-020 (500 ng/ml) or the two treatments combined in the presence or absence of TNFα (20 ng/ml) for 72 hours. (**A**) The MTT assay shows that spirulina alone or NT-020 alone increase proliferation; surprising, the in combination proliferation is decrease compare to control ** p<0.005(compare to media alone). Addition of 20 ng/ml human recombinant TNFα significantly decreased proliferation compare to media alone (***p<0.0001). Only the addition of spirulina alone was able to revert decrease in proliferation †††p<0.05 (compare to TNFα alone). (**B**) Spirulina and NT-020 alone significantly increased the number of BrdU^+^ cells. When added together spirulina and NT-020 cause a significant decrease in proliferation ** p<0.005 ***p<0.0001 (compare to media alone). The addition of TNFα significantly decreased proliferation compare to media alone (***p<0.0001). Spirulina alone was able to prevent the decrease in the number of BrdU^+^ cells. ††† p<0.0001 (compare to TNFα alone).

We next examined the effects of the most effective dose of spirulina (125 ng/ml) and or NT-020 in the presence or absence of TNFα to determine if the effects observed in vivo to promote neurogenesis would be observed in vitro. In Figure 7A a significant effect was found by MTT assay (F_(7,57)_ = 59.83, p<0.0001). It can be observed that spirulina and NT-020 both increase proliferation of human neural stem cells in culture at baseline when measured with the MTT assay. When TNFα was added to the media this reduced the proliferation by approximately 40%, however spirulina, but not NT was able to block this effect in vitro. In the human neural stem cells as opposed to the hematopoetic stem cells there was no additive effect of spirulina and NT-020. To determine if this effect was also observed with a more direct measure of proliferation duplicate cultures were quantified for proliferation using BrdU. As can be observed in Figure 7B a significant effect was found by BrdU (F_(7,47)_ = 252.4, p<0.0001). These data reflect that spirulina and NT-020 increased the numbers of cells dividing in these cultures and that TNFα reduced the proliferation of cells and that spirulina but not NT-020 had an effect to reduce the inhibition by TNFα.

## Discussion

Inflammation is an active process with the purpose of removing or inactivating potentially damaging agents. LPS induces a microbial defense immunity, resulting in the activation of pro-inflammatory pathways, producing inflammatory mediators including cytokines and chemokines. The response to eliminate the ‘danger signals’ does not discriminate and will also cause damage to the uninfected tissue. A failure to regulate inflammation following response to ‘danger signals’ may lead to chronic pathology. We demonstrate that spirulina, a blue-green algae, was able to negate the deleterious effects of LPS induced inflammation on NPC function, by maintaining the proliferative ability of the NPCs in the face of an acute inflammatory insult. Many degenerative diseases and normal aging lead to an inflammatory condition, thus this regulation of the immune response is important under many different kinds of pathological conditions.

The main cell type in the CNS that is responsible for immunity is the microglia. LPS, up-regulates the production of IL-1β by microglia [20]. In our study, we gave only one i.p. injection of LPS, following which we euthanized the rats two days after the LPS insult. At 48 hours after the LPS we did not find an increase in microglia activation as determined by the induction of MHC class II expression. Effects of LPS on microglia are observed at earlier time points following LPS [20] and it is likely that the reason we did not observe an increase was that this phase of activation ends prior to 48 hours post LPS insult. Cytokines such as IL-1β, and TNF-α can also act in an autocrine or paracrine manner to increase the production of IL-1β by microglia [20]. Microglia are the main source of inflammatory cytokines, but astrocytes and neurons can also make inflammatory mediators [21]. Astrocytes can also be stimulated by IL-1β, but not by LPS, to produce TNF-α and IL-6. The secondary response to LPS in astrocytes to microglial produced IL-1β results in increased production of TNF-α beginning about 8 hours, and lasting of 72 hours, and with IL-6 production is still increasing at 72 hour time point [20]. At 48 hours the inflammatory response following LPS has likely transitioned from the initial response of microglia to a secondary response produced by astrocytes. BrdU was injected in the rats at 24 hours after the LPS insult. The decrease in BrdU cell number in the LPS treated rats on the control diet could have been from the initial response of microglia to the LPS or from the secondary response of astrocytes to microglia produced inflammatory mediators. It is not possible, from the results of our study, to determine if the decrease in the number of BrdU^+^ cell was from the initial response of microglia or the secondary response with astrocytes. At 48 hours after LPS there was a measurable astrogliosis in rats on the control diet, but not in rats on the spirulina diet.

Spirulina has been shown to have anti-inflammatory and anti-oxidant effects [13]. The nonsteroid anti-inflammatory drug (NSAID), indomethacin, has been shown able to block the microglia activation and decreased neurogenesis produced by LPS [7]. NSAIDs typically act by blocking cyclooxygenase (COX) activity. The anti-inflammatory properties of spirulina may be attributed to inhibition of COX-2 activity [22] or due the ability to scavenge free radicals [13]. Spirulina has also been shown to enhance innate and adaptive immunity. Immolina, a high molecular weight extract of spirulina, has been shown *in vitro* to be a potent activator of NF-kappa-B in monocytes/macrophages leading to increased IL-1β and TNF-α [23]. The effects of immolina were mediated through the toll like receptor (TLR)-2 and not TLR-4 [24]; therefore it is unlikely that spirulina is acting as a competitive antagonist to the binding of LPS to TLR-4. However, extracting immolina from spirulina might alter the bioactivity of spirulina. Unlike NSAIDs, which act to suppress the immune activation, spirulina may be enhancing the innate immunity resulting in a quicker removal of ‘danger signals’. This effect may be due to priming of the monocytes/macrophages to activation of the TLR [24]. Spirulina has been shown to enhance phagocytosis in macrophages [25], [26], [27] as well as, enhancing cellular and humoral adaptive immunity [25], [28], [29]. To the best of our knowledge, there has been no *in vivo* study to determination of the temporal profile of the immune response to ‘danger signals’ such as LPS in animals fed a spirulina supplemented diet.

We have previously demonstrated that a 4 week spirulina enriched diet in a one-hour middle cerebral artery occlusion (MCAO) could reduce the in infarct size by 70% [30]. We also have found protective effects of spirulina in a rat model of Parkinson's disease. In rats that rats were fed a diet enriched with spirulina prior to a 6-hydroxydopamine (6-OHDA) lesion spirulina helped shift the response of microglia to the injury resulting in improved recovery [31]. At one week after the lesion all the animals irrespective of diet had a similar lesion volume, but the spirulina rats had a more robust immune response. At four weeks after the lesion, the animals that were fed the diet enriched with spirulina had a significant reduction in lesion volume along with a significant decrease in microglia activation [31]. These results suggest spirulina allowed for an early and robust microglia response, which was beneficial in removing the dead and dying cells and remodeling the remaining tissue.

Studies of the nutraceutical formulation NT-020 (a combination of blueberry extract, green tea extract, carnosine, and vitamin D3) has demonstrated that NT-020 is beneficial in supporting repair mechanisms following an injury. In an animal model of stroke, NT-020 was given to the animals for 2 weeks prior to a MCAO and then rats were put back on control diets post injury [4]. The NT-020 treatment was shown to prevent damage from the ischemic insult by 70% when compared to control conditions when examined 2 weeks post stroke. In these same animals it was shown that there was an increase in NPC numbers in both the stem cell niche of the SVZ and at the site of injury in the striatum. This data demonstrated that NT-020 promotes the proliferation of NPC in the niche and migration of these cells to damaged tissues [4]. This result also shows that the effects of NT-020 on cellular migration and health last for at least 2 weeks after the nutraceutical is consumed.

Moreover, in a follow-up study, we found that NT-020 reduced oxidative stress-induced apoptosis of murine neurons and microglial cells *in vitro* [32]. Cultured bone marrow stem cells removed from mice given NT-020 orally for 2 weeks exhibited a dose-related reduction of oxidative stress-induced cell death. This demonstrates that the action of this nutraceutical on stem cells is not dependent on the presence of the formulation as the effect was observed when the cells were cultured in the absence of NT-020 for 3 days. The data presented here suggest that spirulina added to NT-020 can increase the proliferative response of bone marrow and circulating hematopoietic progenitors although the previously mentioned caveat concerning the MTT assay must be considered as this is an indirect measure of proliferation. None the less, our previous studies with spirulina protecting from stroke are very similar to those reported above for NT-020 where we demonstrate using direct measures an increase in NPC proliferation in the NT-020 treatment group.

When the effect of spirulina and NT-020 was examined on neural progenitors in culture we observed an increase in proliferation with both spirulina and NT-020 on these cells at baseline using both the MTT assay and measuring BrdU positive cells demonstrating that the MTT assay, at least in this case, is a reflection of increased numbers of cells. Spirulina, but not NT-020 protected against the effect of TNFα to reduce proliferation of these human neural progenitors in culture. The effect of spirulina to reduce the effect of TNFα in these cultures may be a combination of the effect to increase proliferation at baseline and a direct interaction with the effects of TNFα. Additional studies would need to be conducted in order to determine the mechanism of this protection in vitro. The effect of NT-020 and spirulina appear to differ as they both have effects on baseline, but only spirulina showed significant effects in the presence of TNFα. In the current study, we demonstrate that spirulina maintains regenerative potential of the NPC following an inflammatory insult. We did not examine the differentiation of these cells into neurons following spirulina, however the main impact of aging on NPC's is to reduce proliferation and thus this remains an important finding. It would appear that a diet enriched with spirulina could be beneficial to patient populations at risk for neurodegenerative disease, by promoting a beneficial immune response and limiting some the detrimental effects of an over-active immune system. This study demonstrates an additional beneficial role of spirulina in maintaining the endogenous repair mechanisms of NPCs. This study further demonstrates that spirulina combined with NT-020 can have effects to stimulate proliferation of other stem cell niches such as the bone marrow. This effect on the endogenous repair mechanism of the body may lead to improved health. Although more work is needed to examine these effects in humans, our data may have clinical implications insofar as administration of diets enriched in spirulina or spirulina combined with NT-020 may help maintain our natural repair mechanisms.

## Methods

### Animals

All experiments were conducted in accordance with the National Institute of Health Guide and Use of Laboratory Animals, and were approved by the Institutional Animal Care and Use committee of the University of South Florida, College of Medicine. 3 months old male Fisher 344 (F344) rats (Harlan Sprague Dawley, Indianapolis, IN), were pair-housed in environmentally controlled conditions (12:12 h light:dark cycle at 21±1°C) and provided food and water *Ad libitum.*

### Diet preparation and feeding regimens

NIH-31 (TD 00365; Harlan Teklab, Madison, WI) rodent diet was used as the control diet. NIH-31 rodent diet was supplemented with 0.1% Spirulina w/w dry spirulina (Earthrise Co., Petaluma, CA). Animals were allowed to feed ad libitum for 30 days. Beginning on day zero until day 30 when the rats were euthanized.

### LPS and BrdU injections

On day 28 of the diet animals received a single intraperitoneal (i.p.) injection of LPS (1 mg/kg, i.p.) or equal volume of saline. On day 29, two injection of bromodeoxyuridine (BrdU; 5-bromo-2-deoxyuridine, Sigma, St. Louis, MO, USA) at a dose of 50 mg/kg of was administered (i.p.) with the second injection 12 hours after the first. The rats were euthanized, on day 30, 12 hours after the last injection of BrdU.

### Tissue collection and processing

On day 30 all rats were anesthetized with pentobarbital (50 mg/kg, i.p.). The rats were then perfused transcardialy with phosphate-buffered saline (PBS), followed by 4% paraformaldehyde in 0.2 M phosphate-buffered (PB). The brains were postfixed in 4% paraformaldehyde for 12 hours, after which they were transferred into 30% sucrose in PB for at least 16 hours at 4°C. Sagital sections of the left hemisphere were made at 40 µm using a Microm cryostat (Richard-Allan Scientific, Kalamazoo Michigan), Beginning with a random start, sections were collected including ‘bookending’ sections where the hippocampus was absent to ensure that the entire left hippocampus was included in any future analysis. The brain sections were stored in cryoprotectant at −20°C.

### BrdU Immunohistochemistry

BrdU immunohistochemical staining was conducted on free-floating sections on every sixth section for the entire hippocampus beginning with a random start and including sections before and after the hippocampus to ensure that the entire structure was sampled. Sections were pretreated with 50% formamide/2X SSC (0.3 M NaCl, 0.03 M sodium citrate) at 65°C for 2 hours, rinsed in 2X SSC, incubated in 2N HCL for 30 minutes at 37°C, rinsed with borate buffer (pH 8.5), then washed with PBS. This was followed by quenching endogenous peroxidase activity in 0.3% H_2_O_2_ solution in 30% methanol. The tissue were then blocked for one hour in PBS supplemented with 3% normal horse serum (NHS: LAMPIRE Biological Laboratories, Inc. Pipersville, PA; Cat no. 7333400) and 0.25% Triton X-100 (PBS-TS). BrdU was detected with the primary mouse anti-BrdU (1∶100; Roche; Indianapolis IN; Cat no. 11 170 376 001, clone BMC 9318) diluted in PBS-TS. Samples were incubated in Brdu for 18 hours at 4°C. The following day the sections were washed and then incubated for one hour in a biotinylated horse Anti-Mouse IgG (H+L) (Vector Laboratories, Burlingame, CA; Cat no. BA-2001) at a concentration of 1∶200 in PBS-TS. The biotinylated secondary was amplified in Avidin-biotin substrate (ABC kit, Vector Laboratories, Burlingame, CA); and then developed in DAB solution (SIGMA FAST™ DAB (3,3′-Diaminobenzidine tetrahydrochloride) with Metal Enhancer Tablets Sigma, St. Louis, MO; Cat no. D4418). Sections were then mounted onto glass slides and dried overnight. The sections were then counter stained with Hematoxylin QS (Vector Laboratories, Burlingame, CA; Cat no. H-3404). The tissue sections were dehydrated through gradients of ethyl alcohol, and finally xylene. The sections were then coverslipped with Permount Mounting Medium (Thermo Fisher Scientific Inc. Cat no. SP15-500).

### OX-6 Immunohistochemistry

OX-6 immunohistochemical staining was conducted on free-floating sections as described above with the following alterations: Sections were washed three times in PBS, prior to quenching endogenous peroxidase activity in 0.3% H_2_O_2_ solution in 40% methanol for 20 minutes. Additional washes in PBS preceded a one hour blocking step in 10% normal horse serum and 0.3% Triton X-100. A monoclonal antibody directed against the rat major histocompatibility II (MHC II) (RT1B, Becton, Dickinson Pharmingen, San Diego, CA; Cat no 557016) was used at a concentration of 1∶750 in PBS-TS. Incubation in the OX-6 antibody was done for 18 hours at 4°C. This was followed by three washes in PBS supplemented in 3% normal horse serum, before the addition of a biotinylated horse Anti-Mouse IgG (H+L) (Vector Laboratories, Burlingame, CA; Cat no. BA-2001) at a concentration of 1∶300 at room temperature for 1 hour in PBS-TS. The staining proceeded as described for BrdU above.

### GFAP Immunohistochemistry

Glial Fibrillary Acidic Protein (GFAP) immunohistochemical staining was conducted on every 12^th^ section as described above with the following alterations: Staining was done in large trays with a 96 well insert, allowing for 2 brains from each group to be stained simultaneously. All the brains were stained in one batch, to limit any batch to batch variability. This was important as GFAP immunohistochemisty was found to be very sensitive to batch variations. Sections were washed three times in PBS, prior to quenching endogenous peroxidase activity in 0.3% H_2_O_2_ solution in 40% methanol for 30 minutes. Additional washes in PBS preceded a one hour blocking step in 10% normal goat serum (NGS: LAMPIRE Biological Laboratories, Inc. Pipersville, PA; Cat no. 7332500) and 0.3% Triton X-100. Polyclonal Rabbit Anti- GFAP (Dako North America, Inc; Carpinteria, CA; Cat no. Z0334) was used at a concentration of 1∶10,000 diluted in PBS-TS. Incubation in primary antibody was conducted at 4°C for 12 hours. This was followed by three washes in PBS-TS. The secondary antibody, Biotin-SP-AffiniPure Goat Anti-Rabbit IgG (H+L) (Jackson ImmunoResearch Laboratories, Inc. West Grove, PA; Cat no. 111-065-144) was diluted in PBS-TS at a concentration of 1∶10,000. The staining proceeded as described for BrdU above with the exception of the DAB solution which was used without metal enhancement (SIGMAFAST™ 3,3′-Diaminobenzidine tablets, Sigma, St. Louis, MO; Cat no. D4418).

### Immunofluorescence

Tissues were pretreated with 2N HCL for 2 hours at room temperature, washed, and incubated in blocking solution (0.1 M PBS containing 10% NGS and 0.3% Triton X-100) for 1 hour at room temperature. Tissues were then incubated in primary antibodies overnight at 4°C. In addition to rat anti-BrdU (1∶400;Accurate Chemicals, Westbury, NY Cat no OBT003 clone: BU1/75 (ICR1)) one of the following additional primary antibodies was added: anti-GFAP (1∶500; Dako, Carpinteria, CA), Rabbit anti-Iba1 (1∶500; Wako Chemicals USA, Inc., Richmond, VA; Cat no. 019-19741); goat anti C-terminus of human Doublecortin (DCX) (1∶200; Santa Cruz biotechnology, Santa Cruz, CA; cat no. SC-8066). Tissues were then rinsed 3 times in PBS and the appropriate secondary antibody conjugated to an Alexafluor probe (Molecular Probes, Eugene, OR) was applied for 2 hour. Following 6 washes in PBS, tissues were mounted on slides and coverslipped using Vectashield (Vector Labs, Burlingame, CA).

### Light microscopy imaging and design-based stereology

Stereological quantifications were made on a Nikon Eclipse 600 microscope using Stereo Investigator software, version 7 (MicroBrightField, Colchester, VT). A MBF CX9000 (MicroBrightField, Colchester, VT) digital camera with a native resolution of 1600×1200 active pixels was used to visualized samples on a 24 inch Dell 24WFP at 1900×1200 resolution. LEP Biopoint XYZ (Ludl Electronic Products Ltd., Hawthorne, NY) motorized stage was used for XYZ axis movements.

### BrdU

The estimated number of BrdU^+^ cells was determined using the optical fractionator method of design-based stereological cell counting techniques [15]. Every 6^th^ section throughout the left hippocampus was used to estimate the number of BrdU^+^ cells. Outlines of the subgranular zone (SGZ: defined as a two cell diameter band on both sides of the granular cell layer (GCL)) was done using a 10x/0.45 objective. BrdU^+^ cell quantification was conducted using a 60x/1.40 objective. The optical dissectors were 100 µm×100 µm. The grid size was 250 µm×250 µm. These parameters were established to ensure that a minimum of 200 BrdU^+^ cells were counted per brain with an error coefficients of less than 0.07.

### OX-6

The estimated number of OX-6^+^ cells was quantified for every 6^th^ section. The area sampling fraction was set at one as the entire structure was sampled. Using a 10x/0.45 objective the hippocampus was viewed. OX-6^+^ cells found at low power were confirmed using a 40x/0.75 objective to ensure that the cell was not in the first or last 2 µm of the section. The height of the sampling fraction was set at the average found during the BrdU counts. On average only 20 cells were found per brain. The number of cells counted was used to estimate the total number of cells using the optical fractionator formula [15].

### GFAP

Cell number was determined using the optical fractionator method. Outlines of the dentate gyrus, including the SGZ, were done using a 10x/0.45 objective. The optical dissectors were 100 µm×100 µm. The grid size was 500 µm×500 µm. These parameters were established to ensure that a minimum of 200 GFAP^+^ cells were counted per brain with an error coefficients of less than 0.07. Cell number was quantified using a 40x/0.75 objective. The designed based stereological probe, Area Fraction Fractionator (AFF), was used to determine the area fraction of the dentate gyrus occupied with GFAP^+^ staining. The AFF uses the fractionator method, incorporating an optical dissector with an overlaying cavalieri point counting lattice to sample a fraction of the entire structure, in this case the dentate gyrus. GFAP^+^ staining was counted in the Z plane. Such that if a GFAP^+^ particle (process or soma) first appeared after the 2 µm guard, and the GFAP+ particle intersected with point on the cavalieri lattice, the particle was counted. If, in the Z plane, the same or different GFAP^+^ process crossed the point of the lattice it was counted. Therefore it could be possible to have over 100% of the volume of the region of interest to be positive for staining. However, only the north point of the (+) of the lattice was used as an intersection making the point of intersection small. There was no incidences of two process intersect with one cavalieri point. We used the Z plane as the GFAP^+^ process are not planner; therefore at any one focal plane some process will be in focus while other will be out of focus. If a measurement is made at only one focal plan, the measurement could be biased by the selection of that focal plane. AFF, used in this way, does not give a true absolute volume of the GFAP staining, as you could have more than 100%. However, quantitative comparison can be made between groups stained in unison. The region counted, the counting frame, and the grid size for the AFF were the same as used for the optical fractionator. The cavalieri grid spacing was set at 35 µm.

### Laser scanning confocal microscopy

An Olympus FluoView FV1000 laser scanning confocal microscope was used for all immunofluorescence photomicrographs, only linear adjustments (brightness and contrast) were made to the figures. When quantification of percentage of positive cells was determined, Z stacks were created at 1 µm intervals throughout the 40 µm of the sections with a guard region of 2 µm excluded from top and bottom of the Z stack. The Z stacks were rotated in all planes to verify double labeling.

### Cell Culture

Cultures of human neural progenitors were maintained in culture following the supplier's protocol (HNP1, Neuromics, Edina, MN). Briefly, immediately after thawing, cells (4×10^4^ cells/well) were seeded and grown in 96-well plate coated by poly-L lysine in Neurobasal media (GIBCO, CA) containing 2 mM L-glutamine, 2% B27 (GIBCO, CA) and 50 U/ml penicillin and streptomycin for 4 days at 37°C in humidified atmosphere containing 5% CO_2_. Human bone marrow cells or human CD34^+^ cells (All Cells, Inc.) were cultured in 96 well plates (5×10^4^/well) containing 100 µL of complete medium (RPMI 1640 medium supplemented with 5% FCS). These cells were treated for 72 hours with spirulina at a wide range of doses (15 ng/mL to 500 ng/mL) or NT-020 (proprietary blend of blueberry (500 ng/ml), green tea (500 ng/ml), vitamin D3 as 25-hydroxycholcalciferol (5 uM) and carnosine (20 uM)) the previously published effective concentration [14]. Human recombinant TNFα 20 ng/ml (R&D Systems) was added to human neural cell cultures at the same time as the other treatments.

### MTT assay

Five hours before the end of the treatment, 20 µL of MTT solution (MTT kit, Sigma) was added to each well. These plates were then incubated in a CO^2^ incubator at 37°C for 5 hours and the cultured media removed with needle and syringe. 200 µL of DMSO was added to each well with pipetting up and down to dissolve crystals. These plates were put back into the 37°C incubator for 5 minutes, transferred to plate reader and absorbance measured at 550 nm. Data are represented as relative percentage mean proliferation, defined as O.D. reading number of each treated cells normalized to control cells (in the absence of treatment).

### BrdU immunocytochemistry for cell cultures

Next, in order to reveal the proliferative effects on cultured cells, BrdU immunocytoreactivities were assayed. Each 8×10^4^ cells were plated on 8-well plate coated by poly-L lysine (GIBCO, CA) after achieving confluence at day 4 in culture. Cultured cells were treated for 72 hours as above with the addition of 10 µM BrdU, then rinsed with phosphate buffered saline (PBS) and fixed with 4% paraformaldehyde (PFA) for 30 minutes at room temperature. After blocking reaction with 10% normal goat serum (Vector, CA), cells were incubated overnight at 4°C with anti-BrdU monoclonal antibody (1∶100, Becton Dickinson, CA) with 10% normal goat serum. After several rinses in PBS, cells were incubated for 45 minutes at room temperature in FITC-conjugated anti-mouse IgG (1∶1000, Molecular probe, CA). Cells were counterstained with propidium iodide (PI, cell viability assay) then subsequently embedded with mounting medium. Immunofluorescent images were visualized using Zeiss Axiophot 2 and the number of immunopositive cells was counted per high power field view in 25 fields per well selected at random (50,000 µm^2^). In addition, control studies included exclusion of primary antibody and substituted with 10% normal goat serum in PBS. No immunoreactivity was observed in these controls. All studies were conducted in quadruplicates, with n = 100 fields counted per treatment condition. Assessments were performed blindly by an independent investigator.

### Statistical analyses

Data are presented as mean cell number ± SEM. Statistical analysis was performed using an unpaired, two-side *t*-test, or a one-way ANOVA followed by an un-paired t-test. A value of *p*<0.05 was considered to be significant.
